# Identification and characterization of myocardial metastases in neuroendocrine tumor patients using 68Ga-DOTATATE PET-CT

**DOI:** 10.1186/s40644-018-0168-2

**Published:** 2018-09-20

**Authors:** Wolfgang G. Kunz, Ralf S. Eschbach, Robert Stahl, Philipp M. Kazmierczak, Peter Bartenstein, Axel Rominger, Christoph J. Auernhammer, Christine Spitzweg, Jens Ricke, Clemens C. Cyran

**Affiliations:** 1 Department of Radiology, University Hospital, LMU Munich, Marchioninistr. 15, 81377 Munich, Germany; 2 Department of Nuclear Medicine, University Hospital, LMU Munich, Munich, Germany; 3 Department of Internal Medicine IV, University Hospital, LMU Munich, Munich, Germany; 4 Comprehensive Cancer Center (CCC LMU) and Interdisciplinary Center for Neuroendocrine Tumors of the Gastroenteropancreatic System (GEPNET-KUM), University Hospital, LMU Munich, Munich, Germany

**Keywords:** Myocardium, Neuroendocrine tumors, Positron-emission tomography, Multidetector computed tomography

## Abstract

**Background:**

Focal 68Ga-DOTATATE PET lesions within the myocardium of neuroendocrine tumor (NET) patients are observed in clinical practice. We determined the frequency and characteristics of lesions that are consistent with cardiac metastasis and assessed the lesion detection rate of conventional imaging.

**Methods:**

629 patients who underwent 68Ga-DOTATATE PET-CT at a supraregional comprehensive cancer center on NET were included from a consecutive registry. Inclusion criteria were: (1) focal 68Ga-DOTATATE tracer uptake within the myocardium in more than two sequential PET exams, and (2) contrast-enhanced CT. To determine the diagnostic accuracy of conventional CT imaging, a case-control cohort with a ratio of 1:3 was used. PET and CT were independently analyzed by two blinded readers. Cohen’s κ was assessed for interreader agreement. Descriptive statistics were applied for frequencies and characteristics and group comparisons were analyzed using the Fisher’s exact test.

**Results:**

The prevalence of myocardial metastases related to the registry was 2.4% (15 of 629 NET patients fulfilling the inclusion criteria), for a total of 21 myocardial 68Ga-DOTATATE foci detected. Myocardial lesions were most frequently located in the left ventricle (43%) and the septum (43%). No patient demonstrated a pericardial effusion. Patients with myocardial metastases did not differ in demographics, tumor grading, disease stage or circulating tumor markers compared to the overall registry (all *p* > 0.05). Higher Ki67-Indices were observed (*p* = 0.049) for patients with myocardial metastases. Interreader agreement for PET assessment was excellent (Cohen’s κ = 1.0). CT reading showed a sensitivity of 19% (95% confidence interval: 6–43%) at a specificity of 100% (95% confidence interval: 90–100%).

**Conclusions:**

68Ga-DOTATATE PET enables detection of myocardial metastatic lesions in NET patients**.** In contrast, standard morphologic CT imaging provides very limited sensitivity.

## Background

Neuroendocrine tumors (NET) represent a heterogeneous entity of malignant neoplasms that arise from the cells of the endocrine system [1–3]. At initial staging as well as in therapy monitoring, the use of positron emission tomography (PET) has prevailed over conventional imaging assessment due to a significantly higher sensitivity and specificity [4]. In particular for NET, the tracer 68Ga-DOTATATE allows the detection of metastases based on the typical expression of somatostatin receptor 2 (SSTR2) in well-differentiated NET [5].

The heart is a rare but important site of metastasis for many malignant tumors, which can be localized in different cardiac spaces including the myocardium and pericardium [6, 7]. Pericardial metastases often manifest with concomitant pericardial effusion, which facilitates their diagnosis in conventional computed tomography (CT) or magnetic resonance imaging (MRI). Myocardial metastases, however, often remain undetected until autopsy as indicated by the large differences in the frequency of myocardial metastases in autopsies compared to conventional oncologic imaging [7]. Yet, a reliable way of detecting myocardial metastases is of clinical relevance as it may influence treatment decisions [8, 9].

CT as the most frequently used modality in oncologic imaging has limited sensitivity for the detection of myocardial metastases compared to MRI [7]. Moreover, the most frequently applied PET tracer [18F]-fluorodeoxyglucose (18F-FDG) in oncologic imaging shows high physiologic uptake in the myocardium, which masks focal myocardial lesions with altered glucose metabolism. In contrast to 18F-FDG, the tracer 68Ga-DOTATATE only shows a non-specific background-level uptake of the myocardium [10].

We hypothesize that focal myocardial 68Ga-DOTATATE tracer uptake may contribute to the detection of myocardial metastases in patients with NET. We sought to characterize prevalence, clinical and imaging characteristics in these patients based on the registry of a supraregional interdisciplinary comprehensive cancer center on NET.

## Methods

### Study design and population

The institutional review board of the LMU Munich (Ethikkommission der Medizinischen Fakultät der Ludwig-Maximilians-Universität München) approved this retrospective study, which was conducted according to the Helsinki Declaration of 2013, and waived requirement for informed consent. Based on a prospectively collected NET patient registry at the Interdisciplinary Center for Neuroendocrine Tumors of the Gastroenteropancreatic System (GEPNET-KUM), our initial cohort consisted of 629 consecutive patients who underwent 68Ga-DOTATATE PET-CT between March 2012 and March 2017.

Out of this cohort, we included all subjects with

1. focal myocardial 68Ga-DOTATATE tracer uptake in at least two sequential PET exams, and
2. contrast-enhanced CT.

We excluded patients with

1. non-enhanced CT, and
2. non-diagnostic quality of PET or CT.

### PET-CT and MRI examination protocol

All patients underwent 68Ga-DOTATATE PET-CT (Biograph 64; Siemens Healthcare) 60 min after intravenous injection of a median 223 MBq (standard deviation 22 MBq) of 68Ga-DOTATATE. First, contrast-enhanced CT scans (1.5 mL of iopromide [Ultravist-300; Bayer Healthcare] per kilogram of body weight) were obtained for anatomic localization. Subsequently, the PET scan was acquired by static emission data for 3 min per bed position. PET images were reconstructed using an iterative algorithm (ordered-subset expectation maximization: 4 iterations, 8 subsets). Contrast-enhanced CT data were reconstructed with a slice thickness of 2.0 mm (axial). The reconstructed PET, CT, and fused images were analyzed on the manufacturer’s imaging software (syngo.via; Siemens Healthcare; Forchheim, Germany). Liver MRI, which was routinely performed on 1.5- or 3.0-T scanners (MAGNETOM Aera, MAGNETOM Avanto; Siemens Healthcare; Forchheim, Germany) as a part of the complementary NET staging for the detection of liver metastases, was used to crosscheck for possible heart metastases in cases when the heart or parts of it were included in the field of view. The standard liver MRI imaging protocol consisted of T1-w fast spin echo (FSE) sequences in- and opposed phase, a T2-weighted single-shot FSE sequence without fat saturation (fs), and a breath-hold T2-weighted FSE sequence with fs. We intravenously administrated the contrast agent Gd-EOB-DTPA (Primovist; Bayer Healthcare; Leverkusen, Germany) (0.1 mL of a 0.25 mmol/mL solution per kilogram of body weight) and used a dynamic T1-weighted gradient echo sequence (volumetric interpolated breath-hold examination [VIBE]) with fs. This protocol was not optimized for cardiac imaging. Only 5 patients underwent an additional dedicated cardiac MRI protocol.

### Definition of myocardial metastasis on 68Ga-DOTATATE PET and prevalence

Focal 68Ga-DOTATATE tracer uptake was considered consistent with myocardial metastasis when clearly located in the myocardium and if the uptake was evident on at least two sequential PET exams. Due to the missing necessity for a myocardial biopsy in the overall management of metastasized NET patients, histopathological validation of the myocardial lesions was not available. In a subgroup of five patients, the myocardial lesions could be confirmed by the use of dedicated cardiac MRI examinations. The prevalence of myocardial lesions on 68Ga-DOTATATE PET in NET patients was calculated in relation to the total number of patients treated at our institution in the given time period of this study.

### Diagnostic accuracy of CT for myocardial lesions on 68Ga-DOTATATE PET

To determine the diagnostic accuracy of conventional CT imaging, a case-control-cohort with a ratio of 1:3 was set up including patients without focal myocardial uptake as controls. This design also allows to determine the rate of false-positive findings based on the inclusion of control patients and was chosen to minimize bias during the diagnostic readout [11]. In a randomized and blinded fashion, PET and CT were independently analyzed by two readers, one board-certified in radiology and diagnostic nuclear medicine with 10 years of experience in hybrid imaging (C.C.C.) and one board-certified in nuclear medicine with 15 years of experience in hybrid imaging (A.R.). Interreader agreement was assessed using Cohen’s κ.

### Statistical analysis

We performed all statistical analyses using SPSS Statistics 23 (IBM; Armonk, NY, USA). For the comparison of categorical variables, the Fisher’s exact test was used. Categorical variables are presented as frequency and percentage. All metric and normally distributed variables are reported as mean ± standard deviation; non-normally distributed variables are presented as median (interquartile range, IQR). The Mann-Whitney U test was applied for numeric variables. Normal distribution was assessed using the Kolmogorov-Smirnov test. *P*-values below 0.05 were considered to indicate statistical significance.

## Results

### Patient characteristics

The prevalence of myocardial lesions on 68Ga-DOTATATE PET in relation to the overall patient registry was 2.4%. 15 out of 629 NET patients (age: 65 ± 9; male sex: 60%) with a total number of 21 focal myocardial 68Ga-DOTATATE tracer uptakes fulfilled the inclusion criteria. All focal myocardial uptakes detected were reproducible in follow-up PET exams. There was excellent agreement between the two readers (Cohen’s κ = 1.0). In NET patients with myocardial lesions, the primary tumor was most frequently located in the small intestine (73%). Regarding initial disease stage, distant metastases were frequently observed (73%). Compared to the overall NET registry of our comprehensive cancer center, there were no significant differences of patients with myocardial metastases in patient age, patient sex, tumor grading, initial disease stage, or initial levels of NET tumor markers (all *p* > 0.05). Yet, patients with myocardial metastases demonstrated higher expression of the proliferation marker Ki67-Index compared to the overall registry (*p* = 0.049). None of the patients with myocardial metastases suffered from carcinoid heart disease. In one patient, the myocardial metastasis was operatively resected due to its size with histopathological results consistent with a NET metastasis. The patient characteristics at the initial disease stage are shown in Table 1.

**Table 1:** Characteristics of NET patients with myocardial lesions on 68Ga-DOTATATE PET at the time point of their initial NET diagnosis

	Study population (*n* = 15)	CCC NET registry^a^ (*n* = 629)	*P* value
Age (yrs)	65 (± 9)	63 (± 14)	0.915
Male sex	9/15 (60%)	333/616 (54%)	0.795
Location of primary tumor			
Jejunum/Ileum	11/15 (73%)	182/387 (47%)	N/A
Appendix	1/15 (6.7%)	18/387 (4.6%)	
Colon	2/15 (13%)	27/387 (7.0%)	
Undetermined	1/15 (6.7%)	15/387 (3.9%)	
Tumor grading			
G1	5/10 (50%)	126/255 (49%)	0.353
G2	3/10 (30%)	109/255 (43%)	
G3	2/10 (20%)	20/255 (8%)	
Ki67-Index			
≤ 2%	5/12 (42%)	131/292 (45%)	0.049^b^
> 2–20%	4/12 (33%)	142/292 (49%)	
> 20%	3/12 (25%)	19/292 (7%)	
Disease stage			
Localized	2/15 (13%)	44/227 (19%)	0.743
Regional metastasis	11/15 (73%)	N/A N/A	
Regional metastasis only	2/15 (13%)	21/162 (13%)	1.000
Distant metastasis	11/15 (73%)	113/162 (70%)	1.000
Hepatic	6/11 (55%)	N/A N/A	
Peritoneal	4/11 (36%)	N/A N/A	
Osseous	6/11 (55%)	N/A N/A	
Myocardial	5/11 (45%)	N/A N/A	
Other	2/11 (18%)	N/A N/A	
Chromogranin A (ng/mL)	175 (95–9578)	N/A N/A	
Elevated Chromogranin A	7/10 (70%)	178/312 (57%)	0.526
5-HIAA urine secretion (mg/24 h)	26 (10–80)	N/A N/A	
Elevated 5-HIAA urine secretion	4/6 (66%)	178/312 (57%)	0.701
Carcinoid heart disease	0/15 (0%)	N/A N/A	

Values presented are count/available values (percentage) for categorical, mean and standard deviation or median (interquartile range) for continuous variables. Cut-off values for elevated Chromogranin A and 24-h 5-HIAA urine secretion were 98 ng/mL and 9 mg/24 h respectively. 5-HIAA, 5-hydroxyindoleacetic acid; N/A, not available

^a^ Data are taken from the registry on neuroendocrine tumors (NET) of the comprehensive cancer center (CCC). ^b^Statistical test indicates significant differences between groups

### Detection of myocardial PET lesions by conventional imaging

**Fig. 1:**
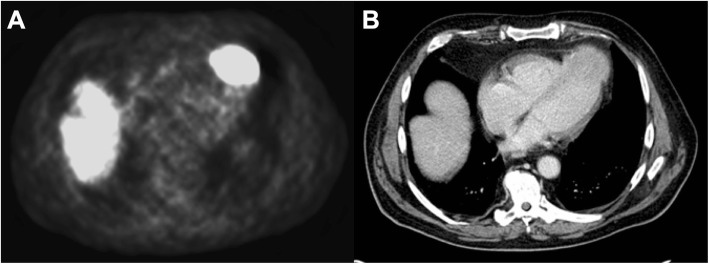
Patient example with a myocardial metastasis located at the apex evident on 68Ga-DOTATATE PET (**a**) and CT (**b**). A 77-year-old male patient with a G3 neuroendocrine tumor of unknown origin with a myocardial metastasis to the apex of the heart. Strong 68Ga-DOTATATE tracer uptake (**a**) correlates to the morphologic mass detected on CT imaging (**b**)

**Fig. 2:**
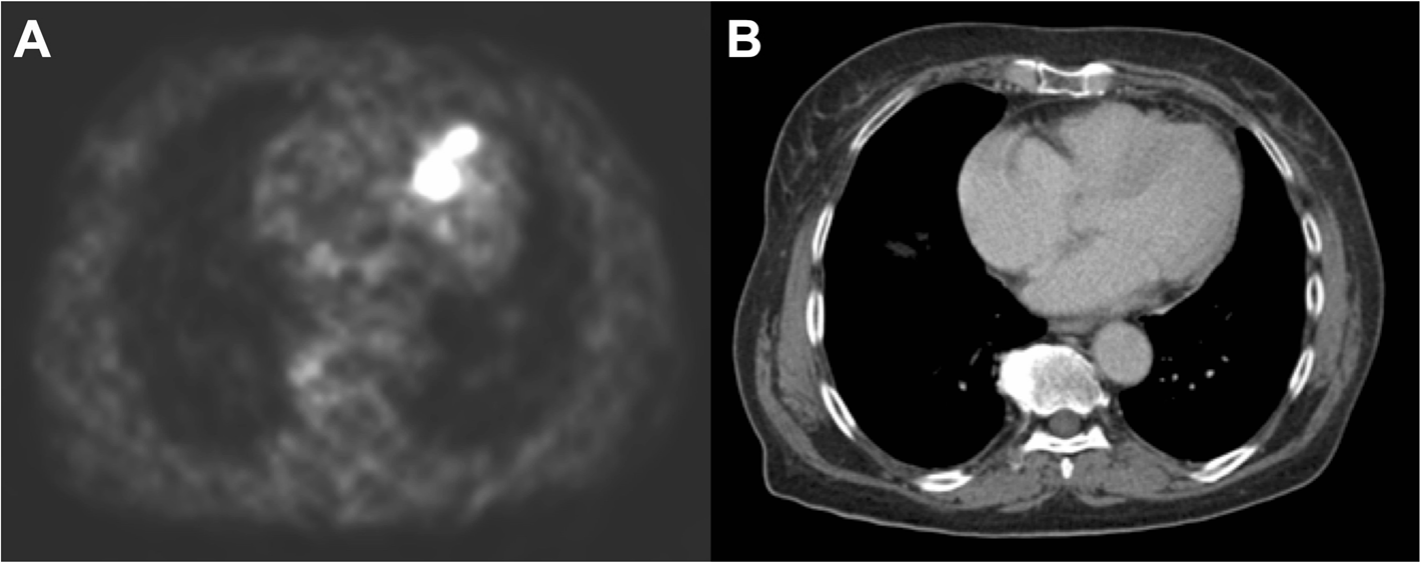
Patient example with a myocardial metastasis detected using 68Ga-DOTATATE PET (**a**) without evidence on CT (**b**). In a 74-year-old female patient with a G2 neuroendocrine tumor of the small intestine, 68Ga-DOTATATE PET (**a**) demonstrates strong focal uptake in the interventricular septum without a morphologic correlate on CT imaging (**b**). This uptake was observed throughout all follow-up examinations consistent with a myocardial metastasis

**Fig. 3:**
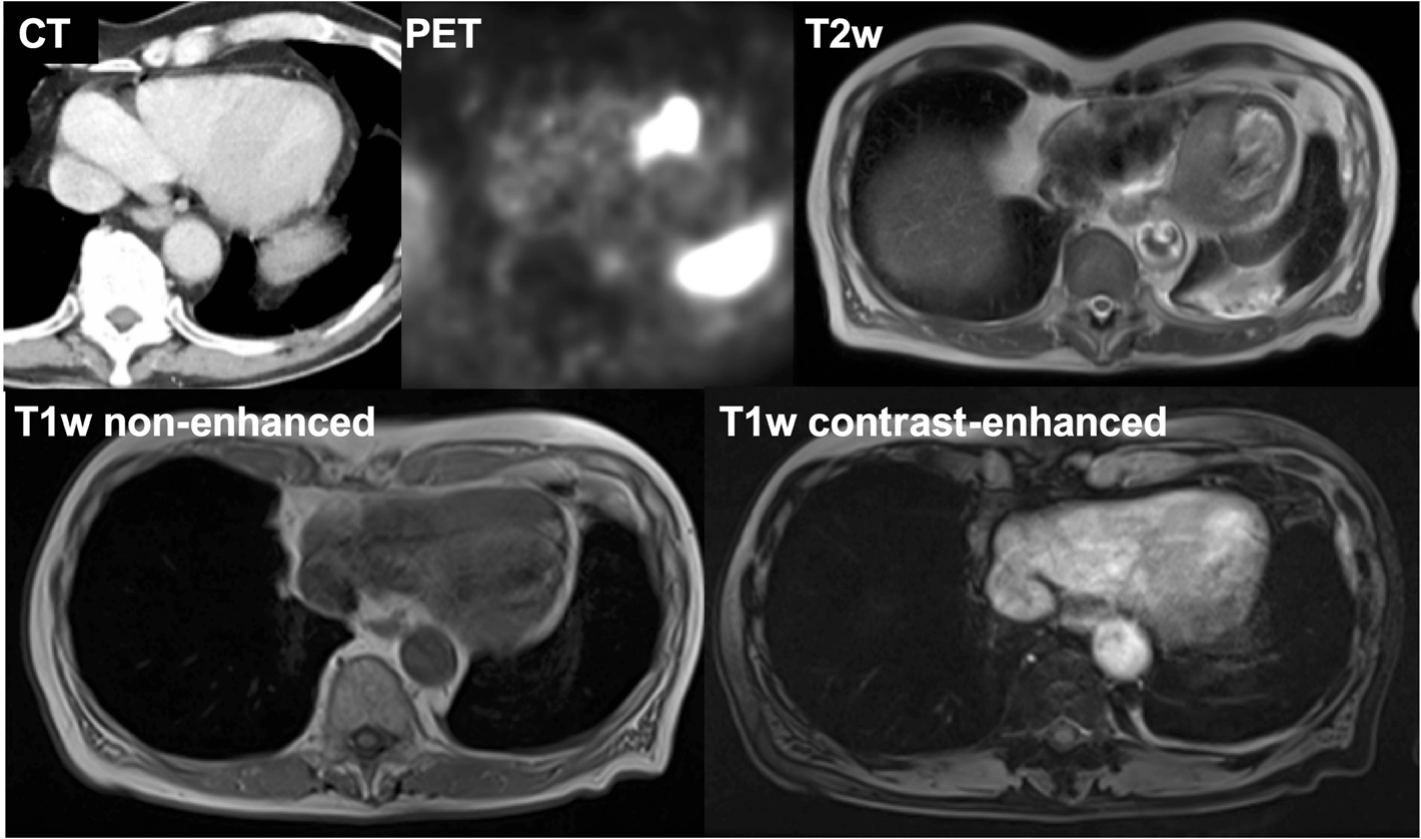
Appearance of a myocardial metastasis on liver MRI performed as part of the complementary staging of the NET disease. A 72-year-old male patient with a G1 neuroendocrine tumor of the small intestine demonstrates a myocardial metastasis in the interventricular septum (evident on CT imaging) with strong 68Ga-DOTATATE tracer uptake in the PET image. The morphologic appearance in the complementary liver MRI is characterized by an intermediate T2w signal and isointense signal on non-enhanced and contrast-enhanced T1w images

CT reading showed a sensitivity of 19% (95% confidence interval: 6–43%) at a specificity of 100% (95% confidence interval: 90–100%) for the detection of myocardial lesions. The diagnostic accuracy measures are shown in Table 2. Liver MRI was part of the complementary NET staging in 14 subjects. Despite the partial field of view and incomplete scanning of the heart, five out of fourteen lesions could be detected with liver MRI. Patient examples are shown in Figs. 1, 2 and 3.

**Table 2:** Diagnostic accuracy of contrast-enhanced CT for myocardial lesions detected on 68Ga-DOTATATE PET in NET patients

	Sensitivity	Specificity	PPV	NPV
All lesions (*N* = 21)				
CT	19 (6–43)	100 (90–100)	100 (40–100)	73 (60–93)

Values presented are percentages with the 95% confidence interval in parentheses. PPV, positive predictive value; NPV, negative predictive value

### Characteristics and conventional imaging appearance of myocardial PET lesions

Myocardial lesions were already detected at initial staging in five patients (33% of study population). In the remaining patients, myocardial lesions were identified a median of six years after the initial diagnosis. The median myocardial lesion SUVmax was 8.6 (relative to the physiologic spleen uptake: 0.386), the median myocardial lesion SUVmean was 4.3 (relative to the physiologic spleen uptake: 0.225). The lesions were most frequently located in the left ventricle (43%) and the septum (43%), no lesions were located in the left or right atrium. If evident, the myocardial lesions most frequently appeared isodense on CT, hyperintense on T2-weighted MR images and isointense on non-enhanced as well as contrast-enhanced T1-weighted MR images. None of the patients demonstrated a pericardial effusion. The characteristics are shown in Table 3.

**Table 3:** Characteristics of myocardial lesions on 68Ga-DOTATATE PET in NET patients

	Patients (*n* = 15) with myocardial lesions (*n* = 21)
Myocardial lesion at baseline	5 (45%)
Time from initial diagnosis to lesion appearance (yrs)	6 (2–10)
Patients with multiple lesions	3 (20%)
Patients with pericardial effusion	0 (0%)
Location	
Left atrial	0 (0%)
Left ventricular	9 (43%)
Septal	9 (43%)
Right atrial	0 (0%)
Right ventricular	3 (14%)
Lesion SUVmax	8.6 (5.2–17.4)
Lesion SUVmean	4.3 (3.7–11.6)
Spleen SUVmax	22.3 (16.4–27.2)
Spleen SUVmean	19.1 (14.7–25.8)
Lesion appearance on CT imaging^a^	
hyper−/ iso−/ hypodense	0 / 3 / 1
Lesion appearance on liver MR imaging^a^	
T2w-hyper- / iso- / hypointense	5 / 0 / 0
T1w-hyper- / iso- / hypointense	0 / 3 / 0
CE-T1w-hyper- / iso- / hypointense	0 / 4 / 0

Values presented are count (percentage) for categorical and median (interquartile range) for continuous variables. SUV, standardized uptake value; T2w, T2-weighted; T1w, T1-weighted; CE-T1w; contrast-enhanced T1-weighted

^a^ If evident on conventional imaging. Liver MRI was only available in 14 subjects

## Discussion

Myocardial metastases detected by the PET component of 68Ga-DOTATATE PET-CT were present in 2.4% of NET patients in our comprehensive cancer center registry. The sensitivity of conventional CT imaging was very low within our study population while yielding very high specificity. None of the patients demonstrated a pericardial effusion, which might have indicated a cardiac spread of the disease. Aside from higher values of the histological proliferation marker Ki67-Index in patients with myocardial metastases compared to the overall registry, no significant differences in patient characteristics were detected. The frequency of myocardial metastases in our patient cohort, the location of metastases, and the time from initial NET diagnosis to diagnosis of myocardial metastasis are in line with previous echocardiographic studies [12–14], with one study also investigating cardiac MRI [14]. The results of our study demonstrate that patients with myocardial metastases from NET had very similar clinical, histological and serological parameters during the initial tumor evaluation. In particular, the tumor grading and the disease stage showed no difference. In line with Calissendorff et al. we also observed myocardial metastases in patients without hepatic metastases [15].

The first reports on myocardial metastases in NET patients based on somatostatin receptor scintigraphy date back to 1996 [16]. Soon after the introduction of 68Ga-DOTATATE PET imaging, reports followed on the detection of focal myocardial uptakes consistent with metastasis [17–19]. We extend the evidence on the diagnostic value of 68Ga-DOTATATE PET by reporting the interreader agreement for myocardial metastasis detection, which was excellent in our study, further supporting 68Ga-DOTATATE PET as a very accurate and robust technique in staging well-differentiated NET. Additionally, we provide data on the repeated detection of the focal myocardial tracer uptakes, which were evident for every myocardial lesion in every follow-up PET exam analyzed**.** Besides 68Ga-DOTATATE, studies also investigated 18-Fluoro-dihydroxyphenylalanin (18F-DOPA) for the detection of myocardial metastases [20, 21], demonstrating the value of functional imaging in the detection of myocardial lesions.

Regarding diagnostic accuracy, we report the low sensitivities of CT imaging for myocardial metastasis detection as evaluated by expert reading of a case-control cohort. We characterized the imaging appearance in contrast-enhanced CT, and in MR imaging of the liver whenever the lesions were depicted and evident. In CT, only the mass effect contributed to the diagnosis as all metastases appeared isodense to the surrounding myocardium. For liver MR imaging, which is often performed as a complementary diagnostic exam in NET patients, T2-weighted sequences were occasionally useful for incidental detection of myocardial metastases. This further supports the use of 68Ga-DOTATATE PET in the staging of NET patients, which has been shown to be superior compared to morphologic imaging in the detection of the primary, the lymphatic as well as the hematogenic spread of the disease [5]. Most importantly, 68Ga-DOTATATE PET significantly impacts therapeutic management in more than 50% of NET patients, including initiation or continuance of peptide receptor radionuclide therapy, medical treatment or referral to surgery. This is primarily driven by improved detection of the primary tumor site or nodal, hepatic and peritoneal metastases [8, 9]. It remains to be determined if the enhanced detection of myocardial metastases impacts patient management.

Carcinoid heart disease (CHD) is a frequent manifestation in NET patients with carcinoid syndrome that has been identified decades earlier than myocardial metastases [22]. CHD significantly contributes to morbidity and mortality of NET patients [13]. It likely results from high levels of circulating vasoactive substances such as serotonin and is characterized by plaque-like, fibrous endocardial thickening involving in particular the right-sided heart valves. This leads to **v**alvular dysfunction and right-sided heart failure. The management of CHD has improved over time, and its prevalence decreased as a consequence of the use of somatostatin analogues [23]. However, 68Ga-DOTATATE PET appears to have no value in the diagnosis of CHD, which is based on echocardiography and circulating biomarkers [23].

Yet, identifying myocardial metastasis may have clinical implications based on the early reported significant overlap with carcinoid heart disease [12]. However, in our study population, none of the patients suffered from carcinoid heart disease at the time that the myocardial lesions were detected, which is in line with a recent report [20]. In accordance with this observation, we detected no difference in circulating tumor markers Chromogranin A or 5-hydroxyindoleacetic acid (5-HIAA) urine secretion, possible biomarkers for CHD [23], between patients with myocardial metastases compared to the overall registry. This further provides support to the hypothesis that CHD and myocardial metastases are truly distinct pathologies.

There are limitations to this study that need to be considered when interpreting the results. First, histopathological validation of the focal myocardial 68Ga-DOTATATE uptake was not available as the risks conferred by myocardial biopsies outweigh the diagnostic yield in the context of metastatic NET patient management. However, repeated focal myocardial PET tracer uptakes are consistent with myocardial metastases. Second, our study population had a limited number of patients, reflecting the comparatively low prevalence of myocardial metastases in NET patients.

## Conclusions

68Ga-DOTATATE PET imaging provides added diagnostic value compared to morphologic CT imaging in the detection as well as in the follow-up of myocardial metastases in NET patients. Further studies are needed to determine the impact on patient management.

## Abbreviations

CT: Computed tomography
MRI: Magnetic resonance imaging
NET: Neuroendocrine tumor
PET: Positron emission tomography
DOTATATE: 1,4,7,10-tetraaza-cyclododecane-1,4,7,10-tetraaceticacid-[Tyr^3^] octreotate

## References

[1] Auernhammer Christoph J, Spitzweg Christine, Angele Martin K, Boeck Stefan, Grossman Ashley, Nölting Svenja, Ilhan Harun, Knösel Thomas, Mayerle Julia, Reincke Martin, Bartenstein Peter (2018). Advanced neuroendocrine tumours of the small intestine and pancreas: clinical developments, controversies, and future strategies. The Lancet Diabetes & Endocrinology.

[2] Yao JC, Hassan M, Phan A (2008). One hundred years after “carcinoid”: epidemiology of and prognostic factors for neuroendocrine tumors in 35,825 cases in the United States. J Clin Oncol.

[3] Ramage JK, Ahmed A, Ardill J (2012). Guidelines for the management of gastroenteropancreatic neuroendocrine (including carcinoid) tumours (NETs). Gut.

[4] Sundin A, Arnold R, Baudin E (2017). ENETS consensus guidelines for the standards of Care in Neuroendocrine Tumors: radiological, Nuclear Medicine & Hybrid Imaging. Neuroendocrinology.

[5] Sadowski SM, Neychev V, Millo C (2016). Prospective study of 68Ga-DOTATATE positron emission tomography/computed tomography for detecting gastro-Entero-pancreatic neuroendocrine tumors and unknown primary sites. J Clin Oncol.

[6] Maleszewski JJ, Anavekar NS, Moynihan TJ, Klarich KW (2017). Pathology, imaging, and treatment of cardiac tumours. Nat Rev Cardiol.

[7] Chiles C, Woodard PK, Gutierrez FR, Link KM (2001). Metastatic involvement of the heart and pericardium: CT and MR imaging. Radiographics.

[8] Ambrosini V, Campana D, Bodei L (2010). 68Ga-DOTANOC PET/CT clinical impact in patients with neuroendocrine tumors. J Nucl Med.

[9] Frilling A, Sotiropoulos GC, Radtke A (2010). The impact of 68Ga-DOTATOC positron emission tomography/computed tomography on the multimodal management of patients with neuroendocrine tumors. Ann Surg.

[10] Tarkin JM, Joshi FR, Evans NR (2017). Detection of atherosclerotic inflammation by 68Ga-DOTATATE PET compared to [18F]FDG PET imaging. J Am Coll Cardiol.

[11] Rothman KJ (2012). Epidemiology: an introduction.

[12] Pandya UH, Pellikka PA, Enriquez-Sarano M, Edwards WD, Schaff HV, Connolly HM (2002). Metastatic carcinoid tumor to the heart: echocardiographic-pathologic study of 11 patients. J Am Coll Cardiol.

[13] Pellikka PA, Tajik AJ, Khandheria BK (1993). Carcinoid heart disease. Clinical and echocardiographic spectrum in 74 patients. Circulation.

[14] Bhattacharyya S, Toumpanakis C, Burke M, Taylor AM, Caplin ME, Davar J (2010). Features of carcinoid heart disease identified by 2- and 3-dimensional echocardiography and cardiac MRI. Circ Cardiovasc Imaging.

[15] Calissendorff J, Maret E, Sundin A, Falhammar H (2015). Ileal neuroendocrine tumors and heart: not only valvular consequences. Endocrine.

[16] Yeung HW, Imbriaco M, Zhang JJ, Macapinlac H, Goldsmith SJ, Larson SM (1996). Visualization of myocardial metastasis of carcinoid tumor by indium-111-pentetreotide. J Nucl Med.

[17] Jann H, Wertenbruch T, Pape U (2010). A matter of the heart: myocardial metastases in neuroendocrine tumors. Horm Metab Res.

[18] Carreras C, Kulkarni HR, Baum RP (2013). Rare metastases detected by (68)Ga-somatostatin receptor PET/CT in patients with neuroendocrine tumors. Recent Results Cancer Res.

[19] Calissendorff J, Sundin A, Falhammar H (2014). (6)(8)Ga-DOTA-TOC-PET/CT detects heart metastases from ileal neuroendocrine tumors. Endocrine.

[20] Noordzij W, van Beek AP, Tio RA (2014). Myocardial metastases on 6-[18F] fluoro-L-DOPA PET/CT: a retrospective analysis of 116 serotonin producing neuroendocrine tumour patients. PLoS One.

[21] Fiebrich HB, Brouwers AH, Links TP, de Vries EG (2008). Images in cardiovascular medicine: myocardial metastases of carcinoid visualized by 18F-dihydroxy-phenyl-alanine positron emission tomography. Circulation.

[22] Roberts WC, Sjoerdsma A (1964). The cardiac disease associated with the carcinoid syndrome (carcinoid heart disease). Am J Med.

[23] Davar J, Connolly HM, Caplin ME (2017). Diagnosing and managing carcinoid heart disease in patients with neuroendocrine tumors: An Expert Statement. J Am Coll Cardiol.

